# Functional Analysis of *Tcl1* Using *Tcl1*-Deficient Mouse Embryonic Stem Cells

**DOI:** 10.1371/journal.pone.0071645

**Published:** 2013-08-05

**Authors:** Tatsushi Miyazaki, Satsuki Miyazaki, Masafumi Ashida, Tomofumi Tanaka, Fumi Tashiro, Jun-ichi Miyazaki

**Affiliations:** Division of Stem Cell Regulation Research, Osaka University Graduate School of Medicine, Suita, Osaka, Japan; Wellcome Trust Centre for Stem Cell Research, United Kingdom

## Abstract

*Tcl1* is highly expressed in embryonic stem (ES) cells, but its expression rapidly decreases following differentiation. To assess Tcl1’s roles in ES cells, we generated *Tcl1*-deficient and -overexpressing mouse ES cell lines. We found that *Tcl1* was neither essential nor sufficient for maintaining the undifferentiated state. *Tcl1* is reported to activate Akt and to enhance cell proliferation. We found that *Tcl1* expression levels correlated positively with the proliferation rate and negatively with the apoptosis of ES cells, but did not affect Akt phosphorylation. On the other hand, the phosphorylation level of β-catenin decreased in response to *Tcl1* overexpression. We measured the β-catenin activity using the TOPflash reporter assay, and found that wild-type ES cells had low activity, which *Tcl1* overexpression enhanced 1.8-fold. When the canonical Wnt signaling is activated by β-catenin stabilization, it reportedly helps maintain ES cells in the undifferentiated state. We then performed DNA microarray analyses between the *Tcl1*-deficient and -expressing ES cells. The results revealed that *Tcl1* expression downregulated a distinct group of genes, including *Ndp52*, whose expression is very high in blastocysts but reduced in the primitive ectoderm. Based on these results, we discuss the possible roles of *Tcl1* in ES cells.

## Introduction

To elucidate the key molecules involved in the pluripotency of mouse embryonic stem (ES) cells, we compared expressed sequence tag (EST) counts between embryonic stem (ES) cells and somatic tissues using digital differential display (http://www.ncbi.nlm.nih.gov/UniGene/info_ddd.html) [1]. The T-cell lymphoma breakpoint 1 gene, *Tcl1*, was one of the genes we identified using this method. This gene is expressed at high levels in ES cells. The normal expression of *Tcl1* in mice is restricted to early embryogenesis [2], fetal tissues (liver, thymus, bone marrow, and yolk sac) [3], developing lymphocytes [4], and adult testis [5], suggesting that it functions in stem cells and progenitor cells. The human ortholog, *TCL1A*, is responsible for T-cell tumors caused by chromosomal rearrangements involving 14q32 [6]. Thus, *Tcl1* may have a positive role in cell proliferation and/or survival, an idea that is supported by the occurrence of T-cell leukemia in mice carrying a *TCL1* transgene under control of the *lck* promoter [7]. On the other hand, an analysis of *Tcl1-*null mutant mice indicated that *Tcl1* is important for the development of preimplantation embryos; a lack of maternally derived *Tcl1* impairs the embryo's ability to undergo normal cleavage and develop to the morula stage, especially *in vitro* [2].

Glover *et al.* [8] identified genes whose expression changes when ES cells are induced to differentiate. *Tcl1* is one of seven genes that showed a rapid decrease in expression concurrent with a decrease in the frequency of undifferentiated cells. Genetic manipulations that affect the undifferentiated state of ES cells are often reported to downregulate *Tcl1* together with other pluripotency-related genes, such as *Dppa3*, *Klf2,* and *Zfp42* [9], [10]. Matoba *et al.* [11] identified *Tcl1* as a downstream target of Oct3/4 using the ZHBTc4 ES cell line, in which the expression of Oct3/4 (encoded by *Pou5f1*) can be downregulated by tetracycline [12]. They showed that Oct3/4 binds to the promoter region of the *Tcl1* gene to activate its transcription, and, using ES cells in which *Tcl1* was knocked down by shRNA, they showed that *Tcl1* is involved in regulating proliferation, but not differentiation. However, the effect of complete loss of the *Tcl1* gene on the state of ES cells has not been reported. In the present study, we generated *Tcl1*-deficient and -overexpressing ES cell lines and compared the undifferentiated phenotypes and gene expression patterns between them.

## Results

### Generation of -deficient ES Cells

We first examined the *Tcl1* expression during ES cell differentiation into trophectoderm using the ZHBTc4 ES cell line, in which the expression of Oct3/4 can be downregulated by tetracycline [10]. As shown in Figure S1, *Tcl1* expression decreased with similar kinetics as *Fgf4*, a well-known target of Oct3/4 [13], consistent with the report by Matoba *et al.* [11] that *Tcl1* is a downstream target of Oct3/4.

**Figure 1:**
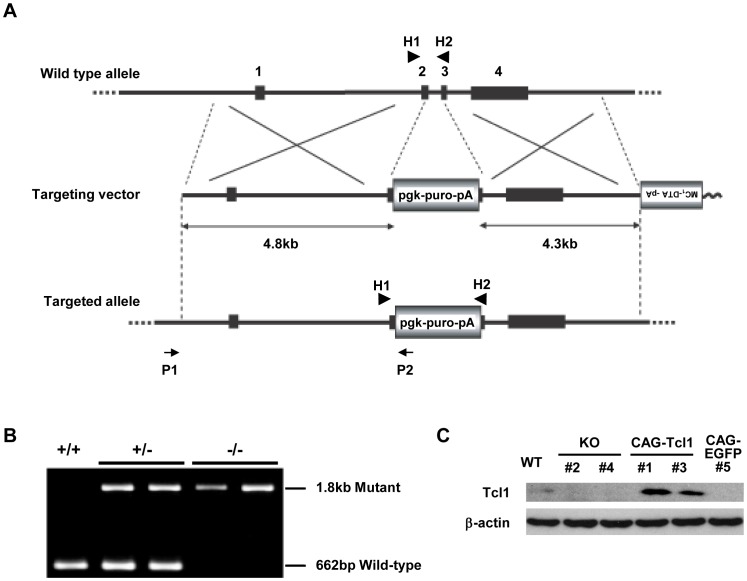
Targeted disruption of the murine *Tcl1* gene. (A) *Tcl1* gene structure and targeting vector. Arrows represent the forward and reverse primers (P1 and P2) used to screen the targeted ES clones, and arrowheads (H1 and H2) represent the primers used to identify homozygous knockout clones. (B) Identification of *Tcl1*+/+, +/−, and −/− ES cell clones by genomic PCR using H1 and H2. (C) Western blot analysis of Tcl1 and β-actin in wild-type (WT), *Tcl1^−/−^* (KO) #2 and #4, *Tcl1^−/−^*(CAG-*Tcl1*) #1 and #3, and *Tcl1^−/−^*(CAG-EGFP) #5 ES cells. *Tcl1^−/−^*(CAG-*Tcl1*) #1 and #3, and *Tcl1^−/−^*(CAG-EGFP) #5 were derived from *Tcl1^−/−^* (KO) #4.

**Figure 2:**
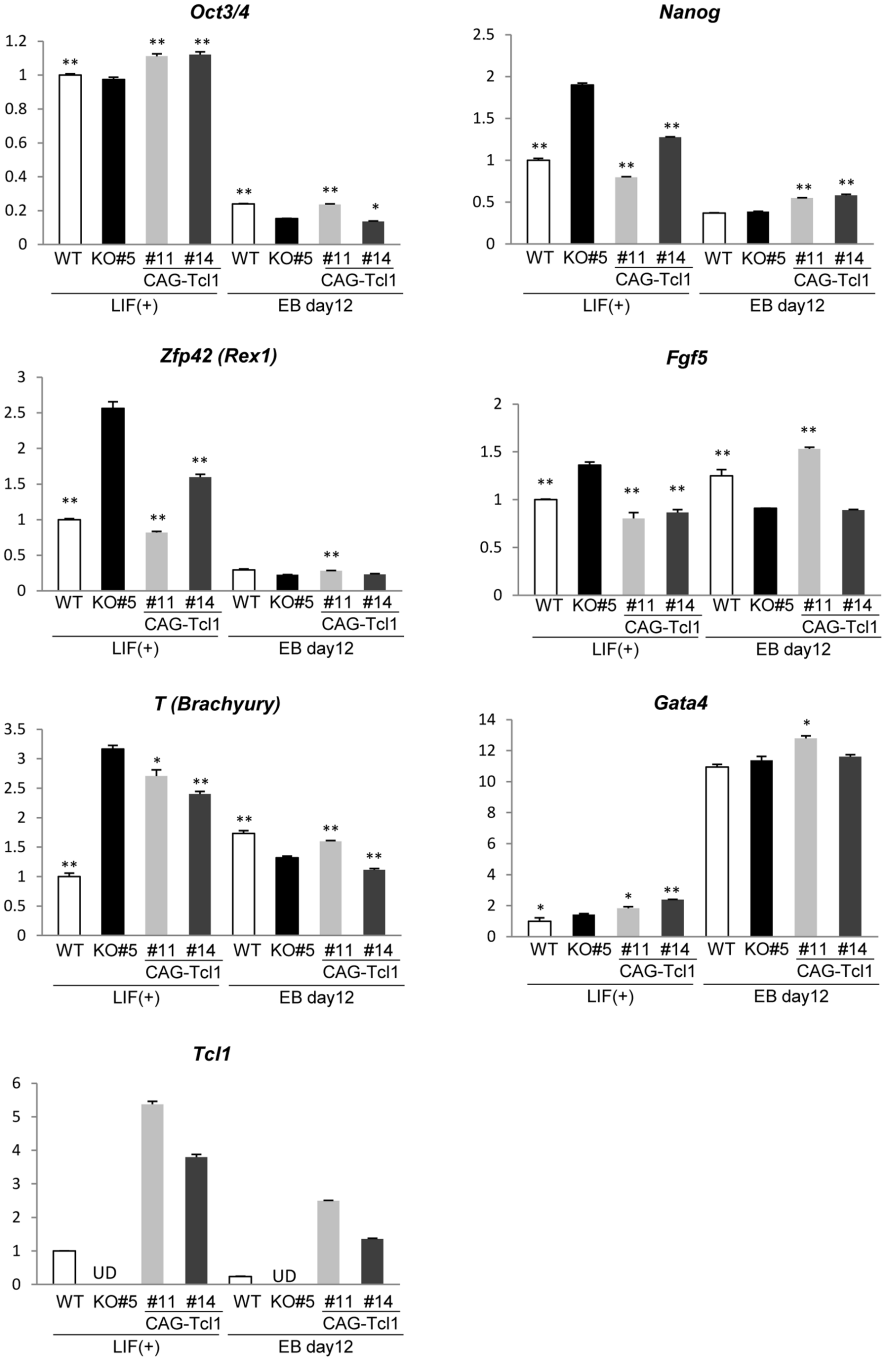
Effects of *Tcl1* deficiency and overexpression on ES-cell differentiation. The expression of representative stem and differentiation markers was examined by real time PCR in ES cells grown in LIF(+) culture (left half of each panel) and with EB formation (right half of each panel). For EB formation, trypsinized ES cells were seeded into a bacterial grade dish, and cultured for 12 days. Values are expressed as mean ± SEM of three technical replicates. ∗*P*<0.05 and ∗∗*P*<0.01 by Student’s t-test. *Tcl1^−/−^* (KO) vs. wild-type (WT) or *Tcl1^−/−^*(CAG-*Tcl1*) cells.

To examine *Tcl1*’s function, we generated *Tcl1*-deficient ES cells. We used a gene-targeting vector in which parts of *Tcl1* exons 2 and 3 were replaced by the PGK-*puro* cassette (Figure 1A) and obtained several *Tcl1^+/−^* clones. Two of these clones were subjected to a high concentration of puromycin, to select for *Tcl1^−/−^* clones (Figure 1B). We chose *Tcl1^−/−^* clone #2, derived from one of the *Tcl1^+/−^* cell clones, and *Tcl1^−/−^* clones #4 and #5, derived from the other clone, for further analysis. Since uniparental disomy accounts for most of the loss of heterozygosity in ES cells [14], it was important to confirm that the phenotypes seen in the *Tcl1^−/−^* clones were due to *Tcl1* deficiency. We first performed karyotype analysis for these *Tcl1^−/−^* clones #2, #4, and #5. More than 60% of the cells from each clone were shown to be karyotypically normal (6/7, 6/7, and 6/10, respectively). We then rescued the *Tcl1* expression in these three *Tcl1^−/−^* ES cell clones by introducing a CAG promoter-driven expression vector containing the *Tcl1* cDNA (CAG-*Tcl1*). The resulting transfectant clones, *Tcl1^−/−^*(CAG-*Tcl1*) #10 derived from *Tcl1^−/−^* clone #2, *Tcl1^−/−^*(CAG-*Tcl1*) #1, #3, and #4 derived from *Tcl1^−/−^* clone #4, and *Tcl1^−/−^*(CAG-*Tcl1*) #11 and #14 derived from *Tcl1^−/−^* clone #5 were chosen for the subsequent experiments. All of these *Tcl1^−/−^*(CAG-*Tcl1*) clones expressed higher levels of *Tcl1* than did wild-type ES cells (Figure 1C and 2). As a control, *Tcl1^−/−^* clone #4 was stably transfected with an EGFP (enhanced green fluorescence protein) expression plasmid (CAG-EGFP), resulting in *Tcl1^−/−^*(CAG-EGFP) #5 and #6.

Neither the *Tcl1^−/−^* ES cells nor the *Tcl1^−/−^*(CAG-*Tcl1*) ES cells showed any apparent changes in cell or colony morphology compared with wild-type ES cells (data not shown). Quantitative real time PCR analysis was performed for wild-type, *Tcl1^−/−^* #5, *Tcl1^−/−^*(CAG-*Tcl1*) #11 and #14 ES cells. The result showed an increase in *Nanog*, *Zfp42* (*Rex1*), *Fgf5*, and *T* (*Bra*) expression in the *Tcl1^−/−^* ES cells, but the espression of *Oct3/4* and *Gata4* was not considerably affected by the *Tcl1* expression (Figure 2 and S2). We also induced the differentiation of these cells by embryoid body (EB) formation and examined the expression of these genes, but did not observe any considerable differences among them (Figure 2).

### Effects of *Tcl1* Deficiency on the Proliferation, Apoptosis, and Differentiation of ES Cells

**Figure 3:**
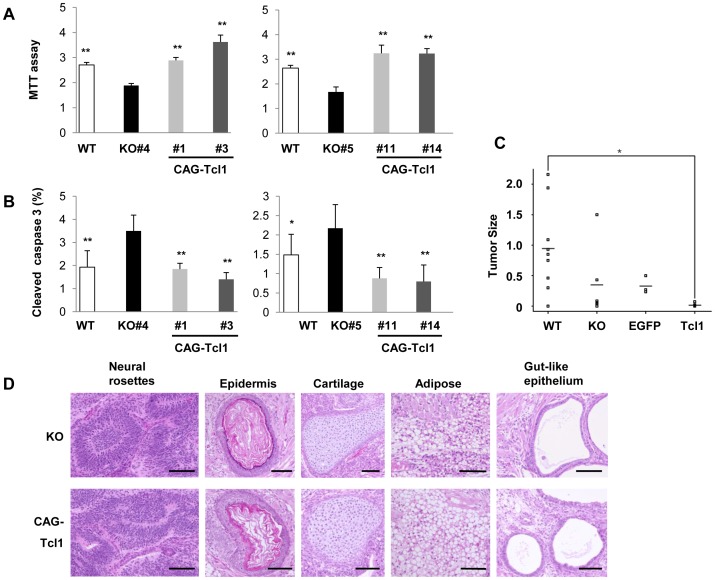
Effect of *Tcl1* expression on ES cell growth. (A) Cell proliferation assay. Cell proliferation between 24 and 48 hours of culture was analyzed by MTT assay. The proliferation rate of *Tcl1^−/−^* (KO) was significantly lower than that of wild-type (WT) or *Tcl1^−/−^*(CAG-*Tcl1*) cells. Values are expressed as means ± SD (left panel, n = 12 each; right panel, n = 8 each). Difference from *Tcl1^−/−^* (KO) ES cells: ∗∗*P*<0.01. (B) The percentage of cleaved caspase 3-positive cells was calculated for 9–13 areas selected at random (left panel, n = 9–13, 6312–14211 nuclei per cell line; right panel, n = 10, 3329–4538 nuclei per cell line). The percentage of cleaved caspase 3-positive apoptotic cells in *Tcl1^−/−^* (KO) cells was significantly higher than that in wild-type (WT) or *Tcl1^−/−^*(CAG-*Tcl1*) cells. Values are expressed as means ± SD. Difference from *Tcl1^−/−^* (KO) ES cells: ∗*P*<0.05, ∗∗*P*<0.01. (C) Teratoma formation of wild-type (WT) (n = 9), *Tcl1^−/−^* (KO) #4 (n = 6), *Tcl1^−/−^*(CAG-EGFP) #5 (n = 3), and *Tcl1^−/−^*(CAG-*Tcl1*) #1 (n = 3) and #3 (n = 5) ES cells. The weight of each teratoma is shown in grams. Horizontal bars indicate the mean of each sample. ∗*P*<0.03. (D) Histological analysis of the teratomas derived from *Tcl1^−/−^* (KO) #4 and #5 and *Tcl1^−/−^*(CAG-*Tcl1*) #1 and #3 ES cells. No histological differences were recognizable between *Tcl1^−/−^* (KO) and *Tcl1^−/−^*(CAG-*Tcl1*) ES cells.

Tcl1 is known to augment Akt function through direct interaction [15]. We next asked whether Tcl1 affected Akt function in our ES cell lines. Akt has three isoforms with similar functions and is activated by various growth factors through its translocation to the cell membrane. This translocation is dependent on phosphatidylinositol 3-kinase (PI3K), which phosphorylates D3 phosphoinositide bound to Akt’s pleckstrin homology domain. At the cell membrane, Akt is activated by phosphorylation at Thr308 (in the case of Akt1) by PDK1 and at Ser473. Activated Akt promotes cell proliferation and survival by inhibiting G1 arrest and proapoptotic factors [16], [17]. Therefore, we analyzed the cell growth of the wild-type, *Tcl1^−/−^,* and *Tcl1^−/−^*(CAG-*Tcl1*) ES cells by MTT assay. As shown in Figure 3A, *Tcl1* deficiency reduced the cell proliferation by approximately 30%, while *Tcl1* overexpression clearly reversed it. These effects were observed using two independent *Tcl1^−/−^* clones (#4 and #5) and their stable transfectant clones (#1, #3, #11, and #14).

We also examined the effects of *Tcl1* deficiency on apoptosis by immunostaining cleaved caspase 3 (Figure 3B). The *Tcl1^−/−^* ES clones (#4 and #5) showed 1.8- and 1.5-fold increase in percent cleaved caspase 3-positive cells, respectively, compared with wild-type ES cells. Thus, *Tcl1* overexpression in these *Tcl1^−/−^* ES cells clearly reduced the frequency of apoptotic cells, which was even lower than that in wild-type ES cells.

We next tested the *in vivo* growth capacity of these cells by performing teratoma formation assays (Figure 3C). The *Tcl1^−/−^* ES cells formed smaller tumors than did wild-type ES cells, consistent with their slower growth rate. To our surprise, however, the *Tcl1^−/−^*(CAG-*Tcl1*) ES cells produced only barely recognizable tumors. Considering that the *Tcl1^−/−^*(CAG-*Tcl1*) ES cells showed a similar or even better proliferation rate *in vitro* than did wild-type ES cells, this result might have been owing to an effect of *Tcl1* overexpression on the differentiation capacity or status of the ES cells. Thus, we examined the teratomas derived from the *Tcl1^−/−^* and *Tcl1^−/−^*(CAG-*Tcl1*) ES cells histologically. Within individual tumors, neural rosettes (ectoderm), epidermis (ectoderm), cartilage (mesoderm), adipose tissue (mesoderm), and gut-like epithelium (endoderm) were found, indicative of the differentiation into cells fated for each of the three germ layers (Figure 3D).

### Effect of *Tcl1* on Wnt-β-catenin Signaling in ES Cells

To gain further insight into *Tcl1*’s function, we looked for signaling pathways that might be affected by it. Akt is known to phosphorylate GSK3β in insulin signaling [16], and GSK3β also serves as a component of the canonical Wnt pathway involving β-catenin. In addition, other reports have indicated a functional link between Akt and β-catenin [18], [19]. Since the Wnt pathway has been implicated in maintaining the undifferentiated state of ES cells, we explored whether Tcl1 acts as a bridge between the Akt signaling and Wnt pathways. In canonical Wnt signaling, briefly, when extracellular Wnt is absent, β-catenin is phosphorylated by casein kinase I and GSK3β. Phosphorylated β-catenin is recognized by E3 ligase and targeted for degradation. Upon Wnt-signaling activation, the phosphorylation of β-catenin is inhibited, and the accumulated β-catenin translocates to the nucleus, where it drives the expression of target genes through an association with Tcf/Lef [20].

**Figure 4:**
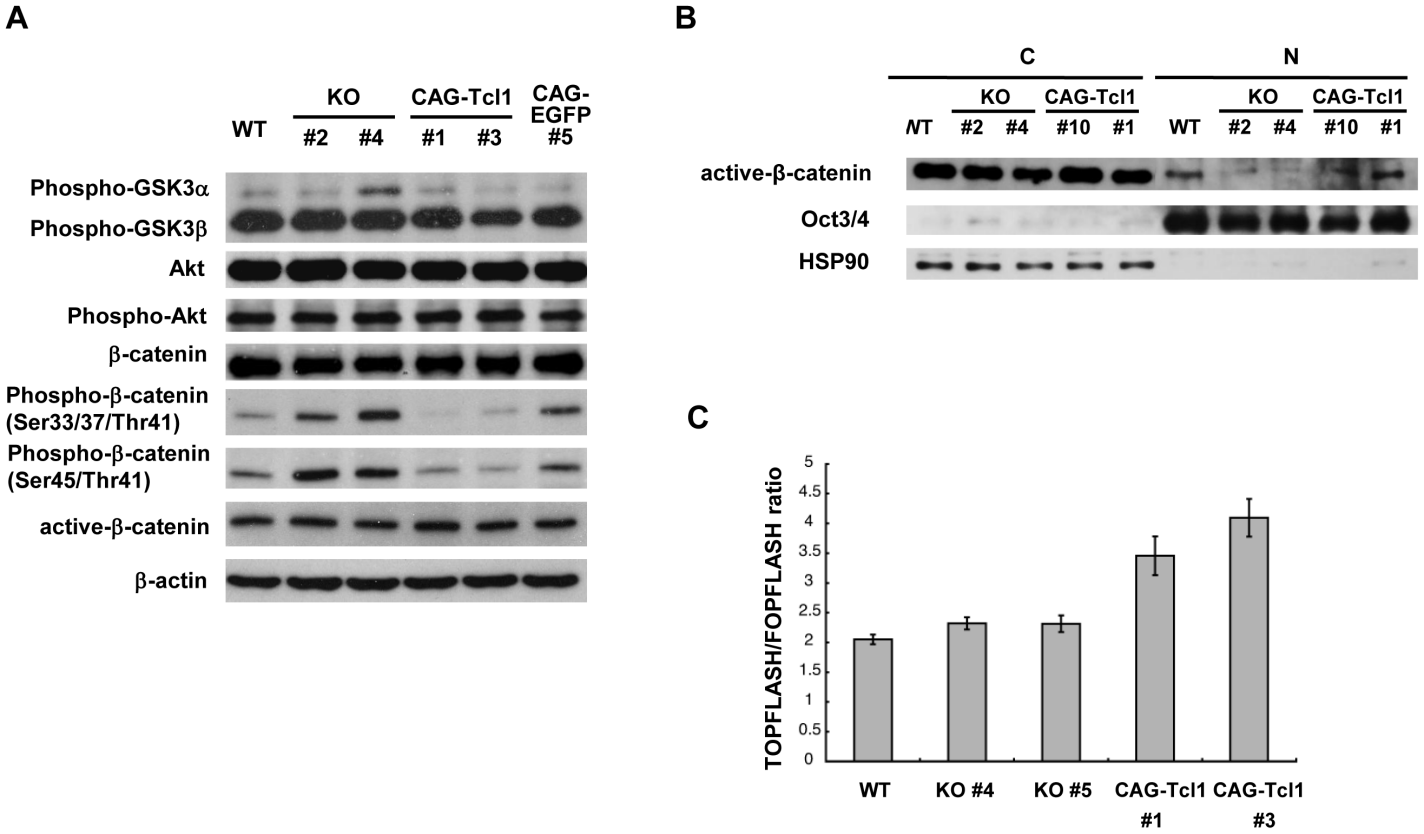
Analysis of Akt and Wnt/β-catenin signaling in *Tcl1-*deficient and -overexpressing ES cells. (A) Western blot analysis of GSK, Akt, and β-catenin in wild-type (WT), *Tcl1^−/−^* (KO) #2 and #4, *Tcl1^−/−^*(CAG-*Tcl1*) #1 and #3, and *Tcl1^−/−^*(CAG-EGFP) #5 ES cells. (B) Western blot analysis of active β-catenin, Oct3/4, and HSP90 in the cytoplasmic (C) and nuclear (N) fractions of wild-type (WT), *Tcl1^−/−^* (KO) #2 and #4, and *Tcl1^−/−^*(CAG-*Tcl1*) #10 and #1 ES cells. *Tcl1^−/−^*(CAG-*Tcl1*) #10 and #1 were derived from *Tcl1^−/−^* (KO) #2 and #4, respectively. Proper fractionation was confirmed by western blotting of Oct3/4 and HSP90, which localize to the nucleus and cytoplasm, respectively. Be8cause active β-catenin levels in the nuclear fractions were much lower than those in the cytoplasmic fractions, active β-catenin in the nuclear fractions was detected by approximately two-fold longer exposure compared with that in the cytoplasmic fractions. (C) TOPflash assay. *P* values of wild-type ES cells (WT) compared with *Tcl1^−/−^*(CAG-*Tcl1*) #1 and #3 ES cells were less than 0.01. *P* values of *Tcl1^−/−^* (KO) #4 and #5 ES cells compared with *Tcl1^−/−^*(CAG-*Tcl1*) #1 and #3 ES cells were less than 0.02.

To examine the effect of *Tcl1* on Wnt-β-catenin signaling, we performed western blot analyses to determine the phosphorylation levels of Akt, GSK3, and β-catenin (Figure 4A). Interestingly, the phosphorylation of β-catenin was dramatically reduced by *Tcl1* overexpression, but the total amount of β-catenin appeared unchanged, indicating there was a reverse correlation between the Tcl1 expression and β-catenin phosphorylation level. At the same time, the Akt and GSK3 phosphorylation levels did not correlate with the β-catenin phosphorylation level, implying that Tcl1 was closely, perhaps directly, involved in regulating β-catenin. We next examined whether *Tcl1* overexpression led to an increase in nonphosphorylated β-catenin in the nuclear fraction, and found that the nonphosphorylated active β-catenin levels were enhanced in the nuclei (Figure 4B).

To measure the canonical Wnt signaling directly, we used a Tcf/β-catenin reporter system, the TOPflash assay [21]. The reporter activity was not affected by the *Tcl1* knockout, but *Tcl1* overexpression enhanced it approximately 1.8-fold compared with wild-type ES cells (Figure 4C). These results suggested that β-catenin signaling is normally repressed in ES cells and is enhanced by *Tcl1* overexpression. We further examined the expression levels of well-known canonical Wnt target genes, such as *c-myc*, *Axin2*, *Lef1*, and *Dll1* (http://www.stanford.edu/~rnusse/pathways/targets.html). Since Tcl1 positively regulates Wnt signaling, the expression levels of these genes might be lower in *Tcl1^−/−^* cells and higher in *Tcl1^−/−^*(CAG-*Tcl1*) cells than in wild-type ES cells. However, our quantitative RT-PCR results showed that the *Tcl1* expression level did not significantly affect these Wnt target genes (data not shown). The only exception was *Gbx2*, a recently identified Wnt-β-catenin signaling target [22], which showed elevated expression in the *Tcl1*-overexpressing ES cells (see below).

### Effects of *Tcl1* Deficiency on the gene Expression Pattern of ES Cells

**Figure 5:**
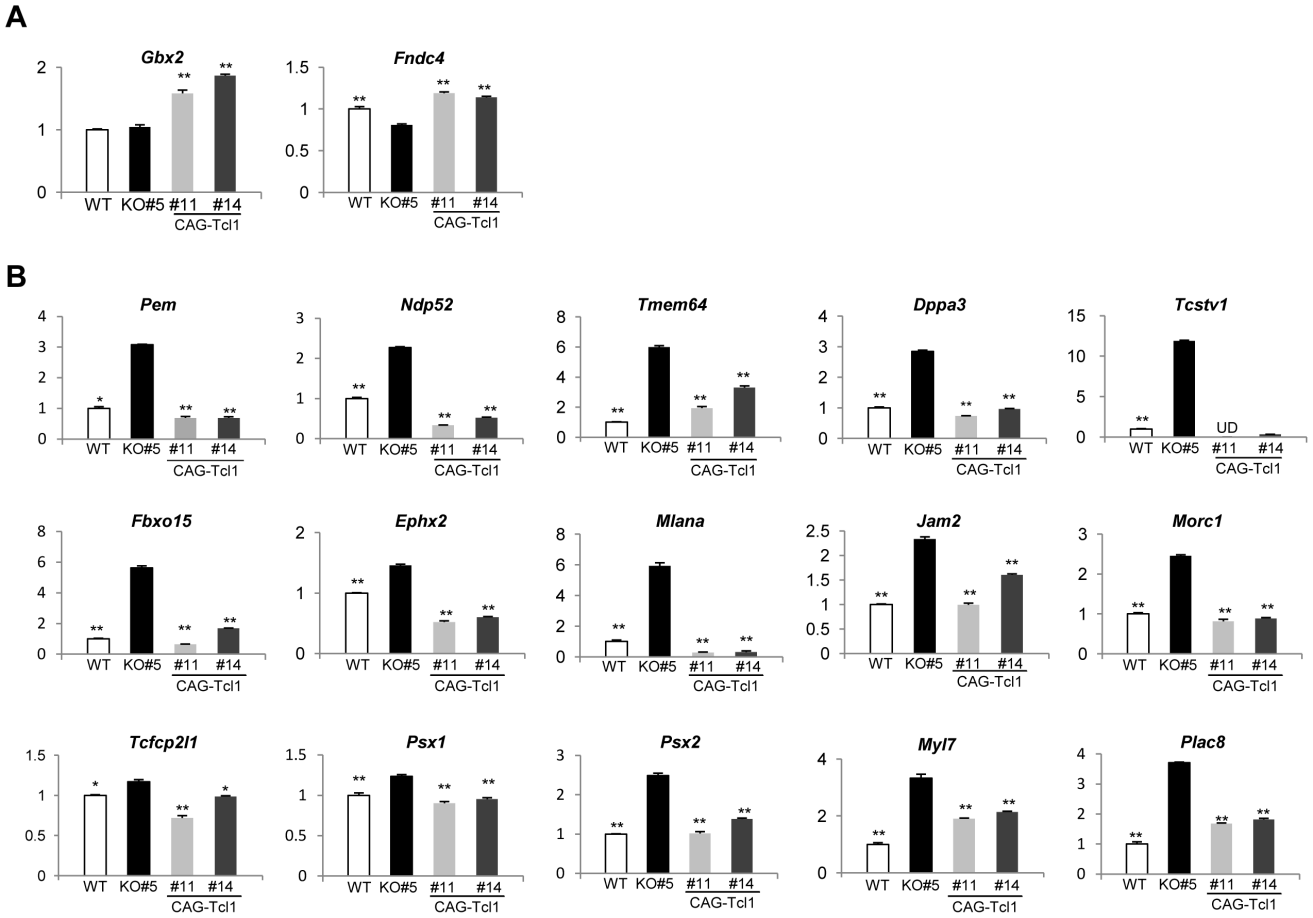
Differentially expressed genes in *Tcl1* *^−/−^* ES cells compared with *Tcl1* *^−/−^*(CAG-*Tcl1*) and wild-type ES cells. (A) Real time PCR analysis of 2 genes that were shown to be upregulated by *Tcl1* expression in DNA microarray analysis. (B) Real time PCR analysis of 16 genes that were shown to be downregulated by *Tcl1* expression in DNA microarray analysis. Expression of these genes were compared among wild-type (WT), *Tcl1^−/−^* (KO) #5, and *Tcl1^−/−^*(CAG-*Tcl1*) #11 and #14 ES cells. *Tcl1^−/−^*(CAG-*Tcl1*) #11 and #14 were derived from *Tcl1^−/−^* (KO) #5. Values are expressed as means ± SEM of three technical replicates. Difference from *Tcl1^−/−^* (KO) ES cells: ∗*P*<0.05, ∗∗*P*<0.01.

To our knowledge, the Wnt targets in ES cells have not been systematically investigated, and there are reports of Tcf/Lef-independent targets in some other cell types. To find genes that respond to *Tcl1* overexpression in ES cells possibly through Wnt/β-catenin signaling, we compared the gene expression profiles by dual-channel DNA microarray analysis between *Tcl1^−/−^* #4 and *Tcl1^−/−^*(CAG-*Tcl1*) #1, between *Tcl1^−/−^*(CAG-EGFP) #6 and *Tcl1^−/−^*(CAG-*Tcl1*) #4, between *Tcl1^−/−^* #4 and wild-type ES cells, and between *Tcl1^−/−^* #5 and wild-type ES cells. The genes whose expression levels were consistently affected by *Tcl1* expression by more than 1.7 fold were listed in Table 1. We found 16 genes (*Pem, Ndp52, Tmem64, Dppa3, Tcstv1, Fbxo15, Ephx2, Mlana, Zfp42, Jam2, Morc1, Tcfcp2l1, Psx1, Psx2, Myl7,* and *Plac8*) that were consistently downregulated by *Tcl1* expression. On the other hand, only two genes, *Gbx2* and *Fndc4,* showed elevated expression in *Tcl1*-expressing cells compared with *Tcl1*-deficient cells and the elevation of their expression by *Tcl1* was 2.7 and 2.2 fold, respectively. Quantitative RT-PCR analysis was performed for these affected genes, using *Tcl1^−/−^* #5, *Tcl1^−/−^*(CAG-*Tcl1*) #11, *Tcl1^−/−^*(CAG-*Tcl1*) #14, and wild-type ES cells. As shown in Figure 5A, the expression of *Gbx2* and *Fndc5* was significantly upregulated by *Tcl1* overexpression, although the expression levels of the *Gbx2* gene were not considerably different between *Tcl1^−/−^* #5 and wild-type ES cells. All of the 16 genes were shown to be downregulated by *Tcl1* expression in agreement with the DNA microarray data (Figure 5B; see Figure 2 for *Zfp42*).

**Table 1:** Representative genes from microarray analysis.

Genes expressed higher in *Tcl1*-expressing ES cells*				
Gene Name	SystematicName	Exp. 1**	Exp. 2***	Exp. 3****
*Gbx2*	NM_010262	2.1	3.7	2.4
*Fndc4*	NM_022424	2.6	2.3	1.7
**Genes expressed higher in ** ***Tcl1*** **-deficient ES cells** *				
**Gene Name**	**Systematic** **Name**	**Exp. 1** **	**Exp. 2** ***	**Exp. 3** ****
*Pem (Rhox5)*	NM_008818	8.4	25.7	2.1
*Ndp52 (Calcoco2)*	AK010816	13.4	6.5	2.9
*Tmem64*	NM_181401	5.1	8.4	1.7
*Dppa3 (Stella)*	NM_139218	6.3	5.1	1.8
*Tcstv1*	NM_018756	3.5	3.5	4.2
*Fbxo15*	AF176530	2.7	4.7	2.6
*Ephx2*	NM_007940	4.5	2.5	2.2
*Mlana*	AK020928	2.8	3.1	2.6
*Zfp42 (Rex1)*	NM_009556	2.9	3.2	2.3
*Jam2*	NM_023844	3.9	2.1	2.4
*Morc1*	NM_010816	3.2	3.1	2.1
*Tcfcp2l1*	NM_023755	2.4	3.6	1.8
*Psx1 (Rhox6)*	NM_008955	2.3	3.2	2.0
*Psx2 (Rhox9)*	NM_023894	2.7	2.7	1.9
*Myl7*	NM_022879	2.5	2.8	2.1
*Plac8*	NM_139198	2.8	2.5	1.9

* Genes listed are those which showed more than 2.0-fold differences in Exp. 1 and 2 and more than 1.7-fold differences in Exp. 3 between *Tcl1*-expressing and -deficient ES cells.

** Exp. 1: *Tcl1^−/−^* #4 vs. *Tcl1^−/−^*(CAG-*Tcl1*) #1.

*** Exp. 2: *Tcl1^−/−^*(CAG-EGFP) #6 vs. *Tcl1^−/−^*(CAG-*Tcl1*) #4.

**** Exp. 3: Mean values of relative gene expression in *Tcl1^−/−^* #4 vs. wild-type and that in *Tcl1^−/−^* #5 vs. wild-type.

## Discussion

In the current study, we sought to elucidate the molecular pathways in which *Tcl1* is involved, and the physiological role of *Tcl1* in ES cell management. One well-documented function of Tcl1 is to bind Akt and increase its kinase activity [23]. Important roles of Akt and/or its upstream signal molecule, PI3K, in the self-renewal of ES cells have been reported [24]–[27]. Matoba *et al.* [11] showed that *Tcl1*-downregulation leads to a reduction in Akt phosphorylation in ES cells. Ema *et al.* [28] showed that Krüppel-like factor 5 (Klf5) is essential for the normal self-renewal of mouse ES cells using *Klf5*-knockout ES cells, and that *Tcl1* is downregulated in the *Klf5*-knockout ES cells. They also showed that the Akt phosphorylation is reduced in these ES cells. These reports support the idea that *Tcl1* regulates ES-cell proliferation via Akt phosphorylation. In fact, we observed that *Tcl1* overexpression in ES cells clearly incresed the cell proliferation and reduced the frequency of apoptotic cells as shown in Figure 3. However, these effects of Tcl1 could not be accounted for by an increase of Akt phosphorylation. Our data showed that *Tcl1* expression was not correlated with the global phosphorylation levels of Akt and GSK3β (Figure 4A). Although we do not know the reason for this discrepancy, it should be noted that high-level *Tcl1* expression does not necessarily lead to Akt phosphorylation, because Akt phosphorylation is undetectable in seminomas and CD4^+^CD56^+^ blastic tumors of dendritic cell origin, in which *Tcl1* is highly expressed [2], [29]. In any case, we believe that our *Tcl1-*deficient and -overexpressing ES cells are ideal tools for identifying Tcl1 targets in ES cells.

Interestingly, our data showed that *Tcl1* expression was correlated with the phosphorylation level of β-catenin (Figure 4A) in ES cells. Tcl1 may regulate β-catenin through a hitherto unknown pathway related or unrelated to Akt or GSK3β. β-catenin plays a major role in the canonical Wnt pathway. It was reported that Wnt-pathway activation by 6-bromoindirubin-3′-oxime (BIO), a specific pharmacological inhibitor of GSK-3, helps maintain the undifferentiated phenotype in human and mouse ES cells [30]. However, Wnt’s precise role in mouse ES cells has been debated [31]–[33].

Stimulation of the canonical Wnt pathway through the binding of Wnt ligand to its receptor causes a repression of GSK3 activity, which further inhibits β-catenin degradation, resulting in β-catenin’s recruitment to the cell membrane to associate with E-cadherin or in its localization to the nucleus. Nuclear β-catenin associates with Tcf1 and activates its transcriptional activator function. It was reported that *c-myc*, a downstream β-catenin target, is involved in maintaining the undifferentiated state [34]. However, it is not known to what degree such transcriptional activation contributes to the maintenance of pluripotency in ES cells. In addition, β-catenin binds to Tcf3 and inhibits its repressor function. Because Tcf3 interacts with core pluripotency-associated transcription factors, such as Oct3/4, to repress their transcriptional activity, its binding to β-catenin may stabilize pluripotency [31]. It is possible that different threshold levels of these two action modes operate in ES cells, in which a low level of Wnt signaling is sufficient to direct a derepression of gene expression through the inhibition of Tcf3 repressor function, whereas high levels are required for the activation through Tcf1. The overexpression of *Tcl1* led to a nearly complete loss of β-catenin phosphorylation, but not to a parallel enhancement of Tcf/Lef reporter activity and activation of target genes. The canonical Wnt activity might be actively repressed or maintained in a narrow range in ES cells. In addition, the nuclear partners of β-catenin in stem cells might be different from those in well-studied somatic cells.

As a negative modulator of β-catenin phosphorylation, Tcl1 may well be involved in anterior-posterior determination and gastrulation development, especially considering that many reports point to the importance of β-catenin at this stage [35]–[37]. The poor teratoma formation elicited by the *Tcl1^−/−^*(CAG-*Tcl1*) ES cells may reflect lineage restriction or premature stem cell/progenitor loss, as was shown to occur during the epithelial-mesenchymal transition in mouse embryos expressing dominant stable β-catenin [35]. Further examination of this issue will require a longer incubation of EBs and the use of differentiation protocols aimed at specific lineages, as well as *Tcl1’*s overexpression in mouse embryos.

Here, to find genes that respond to Tcl1 in ES cells, we compared the gene expression profiles by DNA microarray analyses between *Tcl1*-expressing and -deficient ES cells. We found 2 genes that were consistently upregulated and 16 genes that were consistently downregulated by *Tcl1* expression. Thus, Tcl1 seemed to exert repressive rather than stimulative effects on the gene expression of ES cells. These genes downregulated by *Tcl1* expression could be classified into four categories (Table S1): (1) genes involved in placental function (*Pem, Psx1, Psx2, Plac8*), (2) genes whose expression diminishes during the transition from inner cell mass (ICM) to primitive ectoderm (*Ndp52, Dppa3 (Stella), Fbxo15, Zfp42 (Rex1), Tcfcp2l1 (CRTR-1)*), (3) stem cell marker genes (*Dppa3 (Stella), Tcstv1, Fbxo15, Zfp42 (Rex1), Jam2*), and (4) other genes (*Tmem64*, *Ephx2*, *Mlana, Morc1, Myl7*).

It is not clear whether these changes in gene expression were related to the reduced activity of the Wnt pathway. It is possible that Tcl1 affects the gene expression in ES cells via an unidentified pathway. *Tcl1* expression is reported to be fairly high in embryos during the early cleavage stage and to gradually decline toward the blastula stage [2]. Its expression level during the peri-implantation period is yet to be investigated, but major roles of Tcl1 may be repression of the trophectoderm fate and promotion of the transition from ICM to primitive ectoderm. The fact that the genes involved in placental function and the genes whose expression diminishes during the transition from ICM to primitive ectoderm were downregulated in *Tcl1*-expressing ES cells may be consistent with this notion. Interestingly, the latter genes included *Dppa3,* which is considered a defining marker of the mouse ES cell state and is not expressed in epiblast stem cells [38]. Its expression was reported to be heterogeneous in mouse ES cell cultures. It is thought that the *Dppa3*-negative mouse ES cells are more ‘epiblast-like’ but have not been stably committed to this transition since they readily revert to *Dppa3*-positive [39]. Thus, *Tcl1* may be involved in the the metastability and plasticity of ES cells.

## Materials and Methods

### Ethics Statement

Experiments involving animals were carried out in accordance with institutional guidelines under protocols (No. 21-089-4) approved by the Animal Care and Use Committee of the Osaka University Graduate School of Medicine.

### ES Cell Culture and Differentiation

The murine ES cell line, EB3 [40], which was derived from E14tg2a ES cells, was maintained without feeder cells in Glasgow Minimum Essential Medium (GMEM) (Cat#G6148; Sigma-Aldrich, St. Louis, MO) supplemented with 10% fetal calf serum, 100 µM 2-mercaptoethanol, 1% non-essential amino acids (Cat#11140-050; Life Technologies, Carlsbad, CA), 1% sodium pyruvate (Cat#11360-070; Life Technologies), and leukemia inhibitory factor (LIF) (Cat#129-05601; Wako Pure Chem., Osaka, Japan) on gelatin-coated dishes [12]. All assays were carried out before passage 30. For EB formation, approximately 3.0×10^6^ trypsinized cells were seeded into a 10-cmφ bacterial grade dish, and the culture medium was changed every day starting on day 2.

### Targeted Disruption of the *Tcl1* gene

The targeting vector contained two genomic fragments that had been amplified by long PCR using genomic DNA isolated from EB3 ES cells as a template. The oligonucleotides used for PCR were: left arm forward, 5′-GGCAGATTTAAATAGATGTCCACACCTGATCA-3′ (*Swa*I tailed), left arm reverse, 5′-CGCGATATCACCTGGAATTTTTCATTACTCTG-3′ (*Eco*RV tailed); right arm forward, 5′-ATAGACGCGGCCGCCAACGATGAATAACCCA-3′ (*Not*I tailed), right arm reverse, 5′-TTTAGGCGGCCGCTGGATCTCTTTGTTCCTC-3′ (*Not*I tailed). One fragment (left arm) was 4.8-kb long and contained exon 1 and part of exon 2; the other (right arm) was 4.3-kb long and extended from within exon 3 to 2.8-kb downstream of exon 4.

To assemble the targeting vector, the *Xho*I-*Not*I fragment containing the MC1 promoter-driven diphtheria toxin A gene was excised from pMC1-DTA-pA [41] and subcloned into pBluescript II in which the *Sac*II site had been modified to a *Swa*I site. The amplified left arm was digested with *Swa*I and *Eco*RV, ligated to a DNA fragment containing a phosphoglycerate kinase (Pgk) promoter-driven puromycin acetyl transferase gene cassette, and subsequently introduced into the *Swa*I-*Not*I site of the above vector, which harbored a diphtheria toxin A cassette. Finally, the *Not*I-digested right arm was cloned into the *Not*I site of the targeting vector. The targeting vector was linearized by *Swa*I digestion and introduced into EB3 ES cells by electroporation. Two days later, positive selection was started with 1.5 µg/ml puromycin (Cat#A11138-03; Life Technologies). Clones resistant to puromycin were screened for homologous recombination by long and accurate PCR using primers P1, 5′-TCAGCCCATCTTGGCACATCTGGCAGATT3′ and P2, 5′-TACTTCCATTTGTCACGTCCTGCACGACG-3′. Two of the resulting *Tcl1^+/−^* clones were subjected to a high concentration of puromycin to obtain *Tcl1^−/−^* colonies. The PCR primers used to detect the loss of heterozygosity were H1, 5′-AGGAGCCTGATGATGGTGC-3′ and H2, 5′-GGTCTGGGTTATTCATCGTT-3′. Twenty-eight of 32 clones resistant to the high concentration of puromycin (20∼30 µg/ml) were found to be *Tcl1^−/−^*.

### Generation of *Tcl1^−/−^*(CAG-*Tcl1*) ES Cells

The *Tcl1* ORF was amplified by PCR using *Pfx* polymerase (Cat#11708; Life Technologies) with the following primers: 5′-CGGAATTCCATGGCTACCCAGCGGGCACACAG-3′ (*Eco*RI tailed) and 5′-CGGAATTCCGGTCTGGGTTATTCATCGTTGGAC-3′ (*Eco*RI tailed). The product was digested with *Eco*RI and cloned into the multiple cloning site of pCAG-IZ [42]. The entire expression cassette was excised with *Sal*I and *Bam*HI and inserted between two tandem repeats of *loxP* in a pBS246 derivative [43] lacking the *Eco*RI site. Then, *Tcl1^−/−^* ES cells were transfected with the linearized vector by electroporation. After the transfection, ES cells were selected in the presence of 20 µg/ml Zeocin (Cat#R250-01; Life Technologies) for 7 days. As a control, *Tcl1^−/−^* ES cells were stably transfected with an EGFP (enhanced green fluorescence protein) expression plasmid (CAG-EGFP), resulting in *Tcl1^−/−^*(CAG-EGFP).

### Teratoma Formation

For the teratoma-formation assay, 1.0×10^6^ cells in 75 µl PBS were injected subcutaneously into histocompatible F1 adult mice (C57BL/6J×129/Ola). Four weeks later, the mice were sacrificed, and the tumors were weighed. The tumors were fixed in 20% formaldehyde and processed for paraffin embedding. Sections of paraffin-embedded tumors (5-µm thick) were deparaffinized, stained with hematoxylin-eosin, dehydrated, and examined with a microscope.

### Western Blot Analysis

The total protein was extracted from ES cells. Nuclear and cytoplasmic protein fractions were prepared from ES cells using the NE-PER Nuclear and Cytoplasmic Extraction Kit (Cat#78833; Thermo Fisher Scientific, Rockford, IL). Cell lysates or fractions were subjected to SDS-PAGE and blotted onto a polyvinylidene fluoride (PVDF) membrane (Cat#IPVH09120; Merck Millipore, Billerica, MA). The primary antibodies used were: rabbit anti-Akt (Cat#CST9272; Cell Signaling, Beverly, MA), rabbit anti-phospho-Akt (Ser473) (Cat#CST9271; Cell Signaling), rabbit anti-phospho-GSK3α/β (Ser21/9) (Cat#CST9331; Cell Signaling), rabbit anti-phospho-β-catenin (Thr41/Ser45) (Cat#CST9565; Cell Signaling), rabbit anti-phospho-β-catenin (Ser33/37/Thr41) (Cat#CST9561; Cell Signaling), rabbit anti-Tcl1 (Cat#CST4042; Cell Signaling), mouse monoclonal anti-β-catenin (Cat#610153; BD Biosciences, Franklin Lakes, NJ), mouse monoclonal antibody recognizing active β-catenin that is unphosphorylated at Ser37 and Thr41 (mAb 8E7) (Cat#05-665; Merck Millipore), mouse monoclonal anti-HSP90 (Cat#ADI-SPA-830; Enzo Life Sciences, Farmingdale, NY), mouse monoclonal anti-Oct3/4 (Cat#sc-5279; Santa Cruz Biotechnol., Santa Cruz, CA), and horseradish peroxidise (HRP)-conjugated mouse monoclonal anti-β-actin (Cat#ab20272; Abcam, Cambridge, MA). Goat anti-rabbit Ig (Cat#D0448; DakoCytomation, Houston, TX) and goat anti-mouse IgG (Cat#7072; New England BioLabs, Ipswich, MA) antibodies, both conjugated to HRP, were used as the secondary antibodies at a 1∶1000 dilution, and blots were developed using the ECL Western Blotting Detection Kit (Cat#RPN2209; GE Healthcare Life Sciences, Pittsburgh, PA).

### TOPflash and FOPflash Reporter Assays

Twenty-four hours before transfection, ES cells were plated at 5.0×10^4^ cells per well on 24-well plates. The cells in each well were transfected with 250 ng TOPflash or FOPflash vector (Cat#17-285; Merck Millipore) using Lipofectamine 2000 (Cat#11668; Life Technologies), following the manufacturer's protocol. After 24 hours, the luciferase activities were measured in a luminometer (Luminoskan TL Plus; Labsystems Oy, Helsinki, Finland) using the Dual Luciferase Reporter Assay System (Cat#E1910; Promega, Madison, WI), according to the manufacturer's instructions.

### Microarray Analysis

Total RNA was isolated from ES cells by the acid guanidinium-phenol-chloroform (AGPC) method. The quality of the purified RNA was examined with an Agilent 2100 Bioanalyzer (Agilent Technologies, Santa Clara, CA)). Then, 500 ng of verified RNA was used to generate Cyanine 3 (Cy3)- or Cyanine 5 (Cy5)-labeled cRNA with a Low RNA Input Fluorescent Linear Amplification Kit (Cat#PN5184-3523; Agilent Technologies) following the protocols recommended by the manufacturer. The cRNA was purified with RNeasy mini spin columns (Cat#74104; Qiagen, Hilden, Germany), then 0.75 µg each of the Cy3- and Cy5-labeled cRNAs were combined, fragmented, and used for hybridization to the microarray. For the first hybridization, we used RNA from *Tcl1^−/−^* #4 (Cy3) and *Tcl1^−/−^*(CAG-*Tcl1*) #1 (Cy5). For the second hybridization, we used RNA from *Tcl1^−/−^*(CAG-EGFP) #6 (Cy5) and *Tcl1^−/−^*(CAG-*Tcl1*) #4 (Cy3). These two transfectants were also derived from *Tcl1^−/−^* #4 ES cells. The image was quantified using Agilent Feature Extraction Software. Similarly, microarray analysis was performed between *Tcl1^−/−^* #4 and wild-type ES cells and between *Tcl1^−/−^* #5 and wild-type ES cells.

### Primers used for Semi-quantitative RT-PCR

The primers and RT-PCR conditions for *Fgf5*, *T* (*Brachyury*), and *Gata4* were described previously [44]. The primers for *Gapdh* were purchased (Cat#RPP-401; Toyobo, Osaka, Japan; annealing temperature 60°C, 23 cycles). The primers designed for the current study are listed in Table S2.

### Real Time PCR Analysis

Real time PCR was performed on an ABI Prism 7300 Sequence Detection System, using the SYBR Green PCR Core Reagents (Cat#4304886; Applied Biosystems, Foster City, CA) or FastStart Universal SYBR Green Master (Cat#4913850; Roche, Mannheim, Germany) (primer sequences are shown in Table S3). PCR was performed with an initial step of 10 sec or 10 min at 95°C followed by 40 cycles of 5 sec at 95°C and 31 sec at 60°C. The expression levels of targeted genes were normalized to that of β-actin. Statistical analysis was performed by Student′s t-test.

### MTT [3-(4,5-dimethylthiazol-2-yl)-2,5-diphenyltetrazolium Bromide] Assays

ES cells were seeded in 96-well plates and incubated for 24 or 48 hours, after which MTT (Cat#M5655; Sigma, St. Louis, MO) reagent was added to the wells. After 4 hours, when purple precipitates were visible under a microscope, detergent reagent was added to the wells, and the cells were incubated overnight. A microplate reader (Bio-Rad, Hercules, CA) was used to determine the optical density of each well at 550 nm.

### Apoptosis Study

Apoptosis was assessed by cleaved caspase 3 staining (Cat#CST9661; Cell Signaling) and quantifying the percentage of positive nuclei among DAPI (4′, 6-diamidino-2-phenylindole) -positive nuclei.

### Statistical Analysis

Statistical manipulations were performed using the open-source statistical environment R (http://www.r-project.org). The *P* values for the teratoma weights were calculated with exact Wilcoxon rank sum tests in the exacRankTests package, then adjusted with Holm's method. The *P* values for the TOPflash assay were calculated likewise. Quantitative PCR results are presented as the mean ± SEM. Statistical analyses were carried out by Student’s *t*-test. A value of *P*<0.05 was considered statistically significant.

### GEO Accession Number

The microarray data discussed in this publication have been deposited in NCBI’s Gene Expression Omnibus (GEO, http://www.ncbi.nlm.nih.gov/geo/) and are accessible through GEO Series accession number GSE4801.

## Supporting Information

Figure S1 ***Tcl1*** **expression rapidly declines after** ***Oct3/4*** **suppression.** ZHBTc4 ES cells lack both alleles of the *Pou5f1* gene, and contain an Oct3/4 transgene whose expression is suppressed by tetracycline [12]. RNA was extracted before and 24, 48, 72, and 96 hours after tetracycline was added to the ZHBTc4 cell culture. The *Tcl1*, *Fgf4*, *Pou5f1* (*Oct3/4*), and *Gapdh* gene expressions were analyzed by reverse transcription polymerase chain reaction (RT-PCR).
(TIF)

Figure S2 **RT-PCR analysis of** ***Tcl1*** **-deficient and -overexpressing ES cells.** The expression of stem cell and differentiation markers was examined by RT-PCR in wild-type (WT), *Tcl1^−/−^* (KO) #4 and #5, and *Tcl1^−/−^*(CAG-*Tcl1*) #1 and #3 ES cells grown in LIF(+) culture. *Tcl1^−/−^*(CAG-*Tcl1*) #1 and #3 were derived from *Tcl1^−/−^* (KO) #4. The expression of *T* (*Brachyury*) was enhanced in the *Tcl1^−/−^* ES cells grown in LIF(+) culture.
(TIF)

Table S1 **Annotations for genes of interest in the microarray analysis.**
(DOC)

Table S2 **Primers and cycles for semi-quantitative RT-PCR.**
(DOC)

Table S3 **Primers used for real time PCR.**
(DOC)
