# Correction: Functional Analysis of *Tcl1* Using *Tcl1*-Deficient Mouse Embryonic Stem Cells

**DOI:** 10.1371/annotation/cc1568d0-4299-47b9-896b-d1d12b551224

**Published:** 2013-11-19

**Authors:** Tatsushi Miyazaki, Satsuki Miyazaki, Masafumi Ashida, Tomofumi Tanaka, Fumi Tashiro, Jun-ichi Miyazaki

In the Results section, the subheading "Generation of -deficient ES cells" is incorrect. The corrected subheading is "Generation of *Tcl1*-Deficient ES Cells" 

