# Supplementary figures and images for: Functional Analysis of Tcl1 Using Tcl1-Deficient Mouse Embryonic Stem Cells
Source: PLoS One. 2013 Aug 5;8(8):e71645. doi: 10.1371/journal.pone.0071645 (PMC3733782; doi:10.1371/journal.pone.0071645)

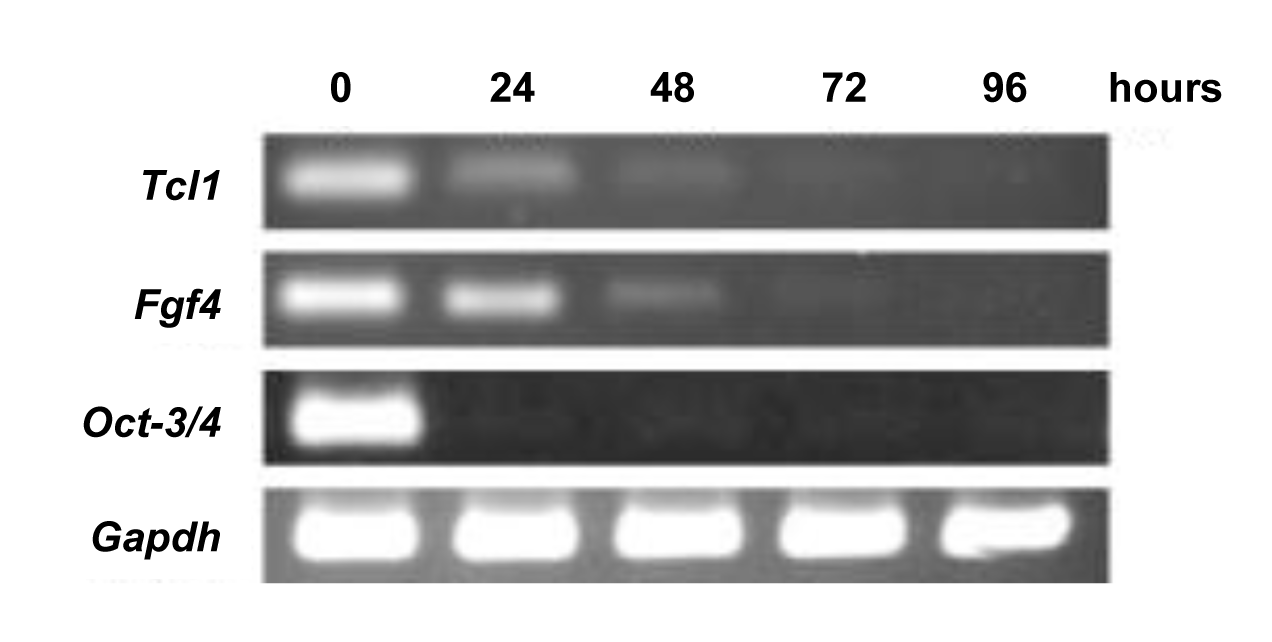

Supplement: Figure S1 — Tcl1 expression rapidly declines after Oct3/4 suppression. ZHBTc4 ES cells lack both alleles of the Pou5f1 gene, and contain an Oct3/4 transgene whose expression is suppressed by tetracycline [12]. RNA was extracted before and 24, 48, 72, and 96 hours after tetracycline was added to the ZHBTc4 cell culture. The Tcl1, Fgf4, Pou5f1 (Oct3/4), and Gapdh gene expressions were analyzed by reverse transcription polymerase chain reaction (RT-PCR). (TIF) [file pone.0071645.s001.tif]

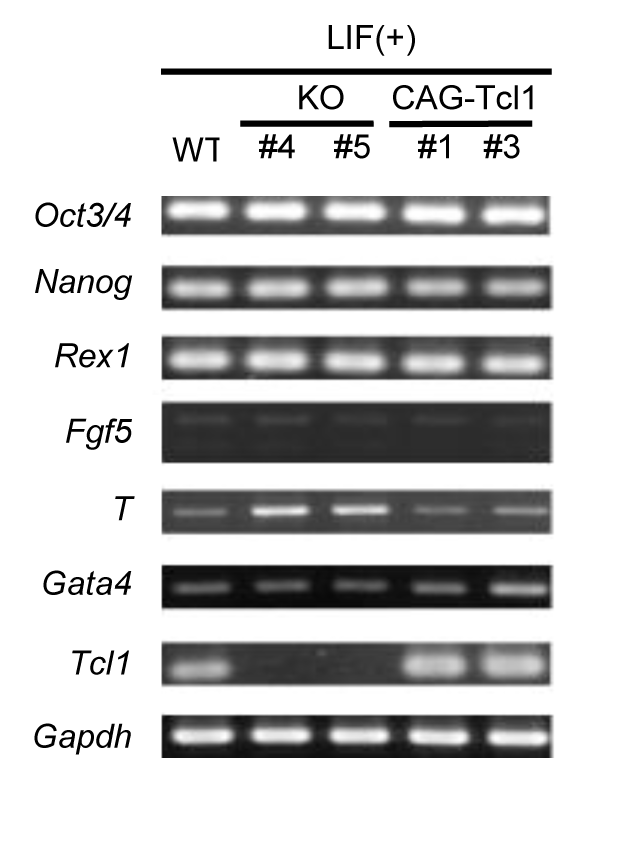

Supplement: Figure S2 — RT-PCR analysis of Tcl1 -deficient and -overexpressing ES cells. The expression of stem cell and differentiation markers was examined by RT-PCR in wild-type (WT), Tcl1−/− (KO) #4 and #5, and Tcl1−/−(CAG-Tcl1) #1 and #3 ES cells grown in LIF(+) culture. Tcl1−/−(CAG-Tcl1) #1 and #3 were derived from Tcl1−/− (KO) #4. The expression of T (Brachyury) was enhanced in the Tcl1−/− ES cells grown in LIF(+) culture. (TIF) [file pone.0071645.s002.tif]
