# Supplementary material for: Functional Analysis of Tcl1 Using Tcl1-Deficient Mouse Embryonic Stem Cells
Source: PLoS One. 2013 Aug 5;8(8):e71645. doi: 10.1371/journal.pone.0071645 (PMC3733782; doi:10.1371/journal.pone.0071645)
Supplement: Table S1 — Annotations for genes of interest in the microarray analysis. (DOC) [file pone.0071645.s003.doc]

| **Table S1. Annotations for genes of interest in the microarray analysis.** | | | |
| --- | --- | --- | --- |
| Gene symbol | Other designations | Annotation | References |
| *Gbx2* | gastrulation and brain-specific homeobox protein 2 | *Gbx2* expression at E7.75 extends posteriorly from a position close to posterior end of the *Otx2* expression domain in all three germ layers. *Gbx2* is expressed in mouse ES cells, but its expression is low in epiblast stem cells. | S1, S2 |
| *Pem* | *Rhox5* (reproductive homeobox 5) | *Pem* is an X-linked imprinted gene and expressed predominantly from the paternally inherited X chromosome. *Pem* is expressed in extraembryonic tissues after implantation and later in primordial germ cells and in adults, in testicular Sertoli cells. Forced expression of *Pem* blocks ES cell differentiation in embryoid bodies. | S3, S4 |
| *Psx1, Psx2* | *Rhox6, Rhox9* | *Pem*, *Psx1*, *Psx2* and several others belong to the *Rhox* (reproductive homeobox ) cluster on chromosome X. | S5 |
| *Plac8* | placenta-specific 8 | *Plac8* is predominantly expressed in trophoblast giant cells and spongiotrophoblasts. | S6 |
| *Ndp52* | *Ndp52l*  *Calcoco2* | *Ndp52* expression is high in E3.5 blastocysts, but low in E7.0 primitive ectoderm. | S7 |
| *Tcfcp2l1* | *CRTR-1* (CP2-related transcriptional repressor 1) | CRTR-1 is expressed in mouse ES cells and in the inner cell mass of E3.5 and E4.5 blastocyst, but is downregulated in E4.75 blastocyst. | S8 |
| *Dppa3* | *PGC7, Stella* | *Stella* is a maternal effect gene required for preimplantation development. Its product binds histone H3K9me2 to prevent the conversion of 5mC to 5hmC in early embryos. *Dppa3* is expressed in mouse ES cells, but its expression is very low in epiblast stem cells. | S9, S10 |
| *Tcstv1* | 2-cell-stage, variable group, member 1 | *Tcstv1* expression is observed in ES cells and in the preimplantation mouse embryo at the 2-cell/4-cell stage. | S7, S11 |
| *Zfp42* | *Rex1* | A putative stem cell marker. [Disruption of the *Zfp42* gene enhances the expression of ectoderm, mesoderm, and endoderm markers compared to wild-type ES cells](http://www.ncbi.nlm.nih.gov/pubmed/19618472). | S12 |
| *Fbxo15* | F-box only protein 15 | *Fbxo15* is a target of Oct-3/4, but is dispensable for ES cell self-renewal. | S13 |
| *Jam2* | *Jam-B* (junctional adhesion molecule B) | *Jam2* expression is highly enriched in undifferentiated ES cells, but is dispensable for normal morphology and pluripotency. | S14 |

**Supplemental References**

S1. Wassarman KM, Lewandoski M, Campbell K, Joyner AL, Rubenstein JL, et al. (1997) Specification of the anterior hindbrain and establishment of a normal mid/hindbrain organizer is dependent on Gbx2 gene function. Development 124: 2923–2934.

S2. Tesar PJ, Chenoweth JG, Brook FA, Davies TJ, Evans EP, et al. (2007) **New cell lines from mouse epiblast share defining features with human embryonic stem cells.** Nature 448: 196–199.

S3. Fan Y, Melhem MF, Chaillet JR (1999) Forced expression of the homeobox-containing gene Pem blocks differentiation of embryonic stem cells. Dev Biol 210: 481–496.

S4. Kobayashi S, Isotani A, Mise N, Yamamoto M, Fujihara Y, et al. (2006) [Comparison of gene expression in male and female mouse blastocysts revealed imprinting of the X-linked gene, Rhox5/Pem, at preimplantation stages.](http://www.ncbi.nlm.nih.gov/pubmed/16431368) Curr Biol 16: 166–172.

S5. Maclean JA, Chen MA, Wayne CM, Bruce SR, Rao M, et al. (2005) Rhox: a new homeobox gene cluster. Cell 120: 369–382.

S6. Galaviz-Hernandez C, Stagg C, de Ridder G, Tanaka TS, Ko MS, et al. (2003) Plac8 and Plac9, novel placental-enriched genes identified through microarray analysis. Gene 309: 81–89.

S7. Zeng F, Baldwin DA, Schultz RM (2004) Transcript profiling during preimplantation mouse development. Dev Biol 272: 483–496.

S8. Pelton TA, Sharma S, Schulz TC, Rathjen J, Rathjen PD (2002) [Transient pluripotent cell populations during primitive ectoderm formation: correlation of in vivo and in vitro pluripotent cell development.](http://www.wikigenes.org/e/ref/e/11839785.html) J Cell Sci 115: 329–339.

S9. Payer B, Saitou M, Barton, SC, Thresher R, Dixon JPC, et al. (2003) Stella is a maternal effect gene required for normal early development in mice. Curr Biol 13: 2110–2117.

S10. Nakamura T, Liu YJ, Nakashima H, Umehara H, Inoue K, et al. (2012) [PGC7 binds histone H3K9me2 to protect against conversion of 5mC to 5hmC in early embryos.](http://www.ncbi.nlm.nih.gov/pubmed/22722204) Nature 486: 415–419.

S11. Zhang W, Walker E, Tamplin OJ, Rossant J, Stanford WL, et al. (2006) [Zfp206 regulates ES cell gene expression and differentiation.](http://www.ncbi.nlm.nih.gov/pubmed/16971461) Nucleic Acids Res 34: 4780–4790.

S12. [Scotland KB](http://www.ncbi.nlm.nih.gov/pubmed?term=Scotland KB%5BAuthor%5D&cauthor=true&cauthor_uid=19618472), [Chen S](http://www.ncbi.nlm.nih.gov/pubmed?term=Chen S%5BAuthor%5D&cauthor=true&cauthor_uid=19618472), [Sylvester R](http://www.ncbi.nlm.nih.gov/pubmed?term=Sylvester R%5BAuthor%5D&cauthor=true&cauthor_uid=19618472), [Gudas LJ](http://www.ncbi.nlm.nih.gov/pubmed?term=Gudas LJ%5BAuthor%5D&cauthor=true&cauthor_uid=19618472) (2009) Analysis of Rex1 (zfp42) function in embryonic stem cell differentiation. Dev Dyn 238: 1863–1877.

S13. Tokuzawa Y, Kaiho E, Maruyama M, Takahashi K, Mitsui K, et al. (2003) Fbx15 is a novel target of Oct3/4 but is dispensable for embryonic stem cell self-renewal and mouse development. Mol Cell Biol 23: 2699–2708.

S14. [Sakaguchi T](http://www.ncbi.nlm.nih.gov/pubmed?term=Sakaguchi T%5BAuthor%5D&cauthor=true&cauthor_uid=16914739), [Nishimoto M](http://www.ncbi.nlm.nih.gov/pubmed?term=Nishimoto M%5BAuthor%5D&cauthor=true&cauthor_uid=16914739), [Miyagi S](http://www.ncbi.nlm.nih.gov/pubmed?term=Miyagi S%5BAuthor%5D&cauthor=true&cauthor_uid=16914739), [Iwama A](http://www.ncbi.nlm.nih.gov/pubmed?term=Iwama A%5BAuthor%5D&cauthor=true&cauthor_uid=16914739), [Morita Y](http://www.ncbi.nlm.nih.gov/pubmed?term=Morita Y%5BAuthor%5D&cauthor=true&cauthor_uid=16914739), et al. (2006) Putative "stemness" gene jam-B is not required for maintenance of stem cell state in embryonic, neural, or hematopoietic stem cells. [Mol Cell Biol 26: 6557–6570.](http://www.ncbi.nlm.nih.gov/pubmed/16914739)
