# Supplementary material for: Functional Analysis of Tcl1 Using Tcl1-Deficient Mouse Embryonic Stem Cells
Source: PLoS One. 2013 Aug 5;8(8):e71645. doi: 10.1371/journal.pone.0071645 (PMC3733782; doi:10.1371/journal.pone.0071645)
Supplement: Table S2 — Primers and cycles for semi-quantitative RT-PCR. (DOC) [file pone.0071645.s004.doc]

| **Table S2. Primers and cycles for semi-quantitative RT-PCR.** | | | |
| --- | --- | --- | --- |
| Gene symbol | Forward primer | Reverse primer | Cycles |
| *Oct3/4* | GGCGTTCTCTTTGGAAAGGTGTTC | CTCGAACCACATCCTTCTCT | 27 |
| *Nanog* | TGCCAGGAAGCAGAAGATGCGGAC | CACTGGTTTTTCTGCCACCGCTTG | 27 |
| *Tcl1* | ATGCCATGGCTACCCAGCGG | CCAAGGTGACATCTTCCTGGC | 28 |
| *Zfp42 (Rex1)* | ATTTTCTGGTGCACACCGGA | CCACCTTCAGCATTTCTTCC | 29 |
| *Fgf4* | ATCGGATTCCACCTGCAGGT | GACACTCGGTTCCCCTTCTT | 28 |
