# Supplementary material for: Functional Analysis of Tcl1 Using Tcl1-Deficient Mouse Embryonic Stem Cells
Source: PLoS One. 2013 Aug 5;8(8):e71645. doi: 10.1371/journal.pone.0071645 (PMC3733782; doi:10.1371/journal.pone.0071645)
Supplement: Table S3 — Primers used for real time PCR. (DOC) [file pone.0071645.s005.doc]

| **Table S3. Primers for real time PCR.** | | | | |
| --- | --- | --- | --- | --- |
| Gene symbol | Forward primer | Reverse primer | Primer bank ID | Product size |
| *β-actin* | AGTGTGACGTTGACATCCGTA | GCCAGAGCAGTAATCTCCTTCT | 6671509a3 | 112 |
| *Nanog* | TTGCTTACAAGGGTCTGCTACT | ACTGGTAGAAGAATCAGGGCT | 31338864a2 | 106 |
| *Oct3/4* | CAGCCAGACCCACCATCTGTC | GTCTCCGATTTGCATATCTCCTG | 7305399a3 | 138 |
| *Fgf5* | AACTCCATGCAAGTGCCAAAT | CGGACGCATAGGTATTATAGCTG | 145966820c3 | 84 |
| *T* | GCTTCAAGGAGCTAACTAACGAG | CGTCACGAAGTCCAGCAAGA | 6678203a1  118130357c1 | 128 |
|  |  |
| *GATA4* | TCAACCGGCCCCTCATTAAG | CACCCTCGGCATTACGACG | 6679953a3 | 117 |
| *Tcl1* | TTAATGTGGCAACTGTACCCC | TCCTCCACGTCTCTAAACTTGAT | 255918187c2 | 95 |
| *Gbx2* | GCAACTTCGACAAAGCCGAG | CCTTGCCCTTCGGGTCATC | 133892275c2 | 180 |
| *Fndc4* | CTTTGATGCCTCTTTCCCCAT | TGACCGTCACATTCACAGGAG | 26378505a1 | 111 |
| *Pem* | ACTCGGAAGAACAGCATGATG | CCCTGGTGCCACTATCCTT | 6679277a1 | 204 |
| *Ndp52* | TGAAAAGTTCATCCCTCGACG | TCCCCGGACTAAACCATCTTC | 154146232c2 | 220 |
| *Tmem64* | GGGGCTACATCGTGCTTAATG | CACATGAGCGATAAAGGTGCC | 225543078c2 | 107 |
| *Dppa3* | GACCCAATGAAGGACCCTGAA | GCTTGACACCGGGGTTTAG | 21218416a1 | 130 |
| *Tcstv1* | GCTGGGCATGGTTTCAAAGG | ACCAGATGGCTGCAAAGACA | Original | 180 |
| *Fbxo15* | TCGTGGGACTGAGCACAACTA | TGACAGATGAGCCTCTAACAAAC | 6456112a1 | 129 |
| *Ephx2* | GCAGCAAGAAGCATCAACCG | AGCCAGTTGTTGGTGACAATG | 118131983c2 | 92 |
| *Mlana* | CTGCTGGTACTGTAGAAGACGA | GGCTCTCACATGAGCATCTTTC | 110625784c2 | 104 |
| *Zfp42* | CCAGCAGCTCCTGCACAC | GCCTATGACTCACTTCCAGGG | Original | 129 |
| *Jam2* | CTGCTACACTACTTGATCGTCG | GTGACTTCTTGACGGTGGTCT | 326537273c1 | 83 |
| *Morc1* | GGCTCCATGAGAATCGGCAAA | GCCATGAAGGTATTGGAACTACA | 7106358c3 | 133 |
| *Tcfcp2l1* | GCTGGAGAATCGGAAGCTAGG | AAAACGACACGGATGATGCTC | 134053938c2 | 75 |
| *Psx1* | TGTTCTGAATAGGCTGGCTCAACTGCGGTACAG | GGAGAGTCGCTCTGGGGAAGAGGC | Ref. | 252 |
| *Psx2* | GGCTGGGAACTATCTGGCTCACCAGCGGACCC | GGGAGAGTTGTTCTCTGTAATCGGTG | Ref. | 253 |
| *Myl7* | TCGGGAGGGTAAGTGTTCC | GTCCGTCCCATTGAGCTTCTC | 114326498c2 | 116 |
| *Plac8* | GCTCAGGCACCAACAGTTATC | GCTGCCACTTGACATCCAAGA | 21105853a1 | 155 |

**Supplemental Reference**

Liu C, Tsai P, García AM, Logeman B, Tanaka TS (2011) [A possible role of Reproductive Homeobox 6 in primordial germ cell differentiation.](http://www.ncbi.nlm.nih.gov/pubmed/22252487) Int J Dev Biol 55: 909-916.
